# Genome-wide association study of posttraumatic stress disorder among childhood cancer survivors: results from the Childhood Cancer Survivor Study and the St. Jude Lifetime Cohort

**DOI:** 10.1038/s41398-022-02110-w

**Published:** 2022-08-23

**Authors:** Donghao Lu, Yadav Sapkota, Unnur A. Valdimarsdóttir, Karestan C. Koenen, Nan Li, Wendy M. Leisenring, Todd Gibson, Carmen L. Wilson, Leslie L. Robison, Melissa M. Hudson, Gregory T. Armstrong, Kevin R. Krull, Yutaka Yasui, Smita Bhatia, Christopher J. Recklitis

**Affiliations:** 1 Unit of Integrative Epidemiology, Institute of Environmental Medicine, Karolinska Institutet, 17177 Stockholm, Sweden; 2 Perini Family Survivors’ Center, Dana-Farber Cancer Institute, Harvard Medical School, Boston, MA 02215 USA; 3 Department of Epidemiology, Harvard TH Chan School of Public Health, Boston, MA 02115 US; 4 Department of Epidemiology and Cancer Control, St. Jude Children’s Research Hospital, Memphis, TN 38105 USA; 5 Center of Public Health Sciences, Faculty of Medicine, University of Iceland, 101 Reykjavík, Iceland; 6 Department of Psychiatry, Psychiatric and Neurodevelopmental Genetics Research Unit, Massachusetts General Hospital, Boston, MA 02114 USA; 7 Public Health Sciences and Clinical Research Divisions, Fred Hutchinson Cancer Research Center, Seattle, WA 98109 USA; 8 Division of Cancer Epidemiology and Genetics, National Cancer Institute, National Institutes of Health, Bethesda, 20892 MD US; 9 Department of Oncology, St. Jude Children’s Research Hospital, Memphis, TN 38105 USA; 10 Department of Psychology, St. Jude Children’s Research Hospital, Memphis, TN 38105 USA; 11 Institute for Cancer Outcomes and Survivorship, School of Medicine, University of Alabama at Birmingham, Birmingham, AL 35233 USA

**Keywords:** Comparative genomics, Psychiatric disorders, Predictive markers

## Abstract

Genetic influence shapes who develops posttraumatic stress disorder (PTSD) after traumatic events. However, the genetic variants identified for PTSD may in fact be associated with traumatic exposures (e.g., interpersonal violence), which appear heritable as well. Childhood cancer survivors (CCS) are at risk for PTSD, but genetic influences affecting cancer are unlikely to overlap with those affecting PTSD. This offers a unique opportunity to identify variants specific to PTSD risk. In a genome-wide association study (GWAS), 3984 5-year survivors of childhood cancer of European-ancestry from the Childhood Cancer Survivor Study (CCSS) were evaluated for discovery and 1467 survivors from the St. Jude Lifetime (SJLIFE) cohort for replication. Childhood cancer-related PTSD symptoms were assessed using the Posttraumatic Stress Diagnostic Scale in CCSS. GWAS was performed in CCSS using logistic regression and lead markers were replicated/meta-analyzed using SJLIFE. Cross-associations of identified loci were examined between CCS and the general population. PTSD criteria were met for 671 participants in CCSS and 161 in SJLIFE. Locus 10q26.3 was significantly associated with PTSD (rs34713356, functionally mapped to *ECHS1*, *P* = 1.36 × 10^–8^, OR 1.57), and was replicated in SJLIFE (*P* = 0.047, OR 1.37). Variants in locus 6q24.3-q25.1 reached marginal significance (rs9390543, *SASH1*, *P* = 3.56 × 10^–6^, OR 0.75) in CCSS and significance when meta-analyzing with SJLIFE (*P* = 2.02 × 10^–8^, OR 0.75). Both loci were exclusively associated with PTSD in CCS rather than PTSD/stress-related disorders in general population (*P*-for-heterogeneity < 5 × 10^–6^). Our CCS findings support the role of genetic variation in PTSD development and may provide implications for understanding PTSD heterogeneity.

## Introduction

Cancer survivors face significant stress at diagnosis and during treatment, as they often face uncertainty, aversive and painful medical procedures, insecure prognosis, and possible death or disfigurement [1, 2]. Childhood cancer survivors (CCS) are particularly vulnerable to long-term psychological effects, as cancer and its treatment may profoundly disrupt their physical and emotional development [3]. Studies indicate that survivors are at increased risk of developing posttraumatic stress disorder (PTSD) compared to their cancer-free siblings [4], and as many as 20% are symptomatic many years after treatment completion [5, 6]. PTSD significantly impacts survivors’ health and quality of life [3]. Therefore, it is important to identify CCS at risk for PTSD early and provide appropriate treatment. Prior research on CCS has identified potential treatment-related risk factors for PTSD, including radiation at a young age (<4 years) and more intensive cancer treatment [4]. PTSD symptoms in early adulthood are more commonly reported by female or unmarried survivors and those with lower socioeconomic status [4, 7]. However, little is known about why some survivors develop PTSD symptoms and others do not.

In the general population, emerging data lend support to a genetic influence on PTSD [8], with a heritability of 38–46% in twin studies including both sexes [9, 10]. An increasing number of genetic markers of PTSD have been identified by the Psychiatric Genomics Consortium (PGC) [11] and in the veteran population [12, 13]. However, the estimated heritability in genome-wide association study (GWAS) is lower, ranging from 5–20% depending on sex and subpopulation [11]. In addition to genetic variations not captured by GWAS, the missing heritability in PTSD GWAS compared to twin studies is likely due to the heterogeneity created by different PTSD measurements and different traumatic events preceding PTSD. A PTSD diagnosis requires both exposure to a traumatic event and development of a constellation of symptoms in response. Although trauma exposures are typically considered external events, interpersonal traumatic events (e.g., childhood abuse and interpersonal violence) have been shown to be significantly heritable [60% in a twin/sibling study and 5.7–12.3% in GWAS] [9, 14] and to overlap with genetic influences on psychopathology, including PTSD [15]. The genetic effects on traumatic events suggest that some risk loci may impact risk for traumatic exposures and some impact risk of developing PTSD after traumatic exposure, whereas other loci may influence both [8]. Moreover, in previous GWAS of PTSD [11–13, 16], not all control participants (those without PTSD) had been exposed to a traumatic event. Loci detected in prior GWAS are those that stand out from the heterogenous traumatic exposures experienced by the entire cohort. In contrast, CCS were all exposed to the same, non-interpersonal traumatic event, and the genetic contribution to childhood cancers is relatively small (8–12%) [17, 18] and therefore less likely to overlap with PTSD psychopathology. This offers a unique opportunity to identify genetic variants specific to risk for PTSD.

Leveraging two unique large cohorts of CCS, we aimed to investigate the genetic contribution to PTSD risk in CCS and advance our understandings of the potentially shared genetic markers in relation to PTSD following other traumatic events.

## Materials and methods

### Study population

A GWAS was conducted using the Childhood Cancer Survivor Study (CCSS) [19]. The CCSS is a retrospective cohort with longitudinal follow-up that enrolled 25,665 survivors of childhood cancer diagnosed between 1970 and 1999 from 31 collaborating centers in the USA and Canada, and enumerates long-term health status, including behavioral and sociodemographic outcomes [20]. Participants were recruited from individuals treated for an initial diagnosis of leukemia, central nervous system (CNS) malignancy, Hodgkin lymphoma, non-Hodgkin lymphoma, kidney cancer, neuroblastoma, soft-tissue sarcoma, or malignant bone tumors at the collaborating centers, diagnosed before age 21, and survived >5 years. Among the original cohort diagnosed between 1970 and 1986 there were 20,267 eligible survivors, with 14,024 (81%) survivors enrolled. The eligible CCSS population is estimated to include about 40–45% of all U.S. 5-year survivors from that time period [20]. Genotype data were available for 5739 (41%) participants who were diagnosed during 1970–1986 and 5149 responded to the follow-up questionnaire assessing PTSD symptoms. After excluding participants who did not complete the PTSD assessment (*n* = 720), first-degree relatives (having identity-by-descent sharing >0.70 as described elsewhere [21]; *n* = 61), and non-European-ancestry [as there were too few to conduct a statistically sufficient analysis; *n* = 384 (84 cases and 300 controls)], 3984 European-ancestry participants were included for analysis. Institutional review boards at each collaborating center approved the CCSS protocol; all study participants provided informed consent.

The St. Jude Lifetime Cohort Study (SJLIFE) [22] was employed as the replication cohort. SJLIFE is a retrospectively identified and prospectively followed cohort study of 5-year CCS treated at St. Jude Children’s Research Hospital (SJCRH). Patients who were treated for a malignancy at SJCRH between 1962 and 2012 and survived ≥5 years are eligible for inclusion. At the time of analysis, SJLIFE has enrolled 5017 survivors; among those, 3006 were whole genome sequenced and 2815 completed PTSD assessment. After excluding the overlapping samples with the CCSS, we included 1467 European-ancestry individuals for analysis. The study was approved by the SJCRH institutional review board and written informed consent was obtained from all participants.

### Ascertainment of PTSD

In the CCSS cohort, we assessed PTSD symptoms specifically related to participants’ childhood cancer experience with the Posttraumatic Stress Diagnostic Scale (PDS). The PDS has been previously validated and yielded an 82% agreement with structured clinical interview for PTSD [23]. As part of the CCSS Follow-up 2 survey, which was administered on average 23.7 (SD 4.6) years after cancer diagnosis, the PDS was sent to all eligible survivors (*n* = 9308) and completed by 7040 (76%). The PDS includes 17 questions covering re-experiencing, avoidance, and arousal symptoms. Frequency of each symptom in the prior month is rated on a 4-point scale from 0 (“not at all or only one time”) to 3 (“almost always”). Symptoms rated >0 were counted as present. Using these scoring criteria, the PDS has been shown to have good internal consistency and test-retest reliability, as well as satisfactory convergent and concurrent validity [24]. Based on the DSM-IV criteria, participants were classified as probable cases of PTSD if they had ≥1 re-experiencing symptom(s), ≥3 avoidance symptoms, and ≥2 arousal symptoms. As the secondary outcome, a total score of PTSD symptoms was calculated by summing the scores of 17 symptom items and converting it to *z*-score for analysis. Subscores for re-experiencing, avoidance, and arousal symptoms were also derived.

In the SJLIFE cohort, the PTSD Checklist Civilian Version (PCL-C) was used on average 21.0 (SD 8.2) years after cancer diagnosis to evaluate PTSD symptoms without reference to a specific traumatic event. The PCL-C includes 17 symptom items on a Likert-scale ranging from “not at all” to “extremely.” A total score was calculated by summing 17 items (range 17–85), and following published standards, those with a score ≥44 were classified as probable cases of PTSD [25]. The PCL-C has been validated in cancer patients [26] and has shown good internal consistency in SJLIFE [7].

### Functional status and psychological adjustment

In the CCSS cohort, functional status was evaluated by using the RAND Health Status Survey Short Form-36 (SF-36) [27]; and psychological adjustment was assessed by using the Brief Symptom Inventory-18 (BSI-18) at the time of PTSD assessment [28]. Consistent with a prior CCSS study [4], functional impairment was defined as *t*-score ≤ 40 on the “role limitations due to emotional health” factor, and psychological distress was defined as *t*-score ≥ 63 on the Global Status Index scale or *t*-score ≥ 63 on any two Depression, Anxiety, and Somatization factors.

### Genotyping, imputation, and quality control

In the CCSS cohort, DNA was extracted using standard methods from blood, saliva, or buccal cells, which were collected at a mean age of 31.7 (SD 8.5) years and on average 23.0 (SD 5.8) years after cancer diagnosis. DNA was sequenced using the Illumina HumanOmni5Exome array (San Diego, CA). As described elsewhere [21] and in Supplementary Methods, information on 26,135,905 single-nucleotide polymorphisms (SNPs) and small insertions or deletions was available after imputation. In the SJLIFE participants, DNA were extracted from blood samples obtained at a mean age of 30.8 (SD 8.8) years and on average 22.1 (SD 8.7) years after cancer diagnosis. Whole genome sequencing was done using the Illumina HiSeq X10 sequencers (30× average coverage). After quality control, information on approximately 84.3 million autosomal single-nucleotide variants and small insertions and deletions (indels) was available.

### Statistical analysis

#### GWA analysis

In CCSS participants, GWAS was performed using logistic regression for the analysis of PTSD cases and linear regression for PTSD symptom score in PLINK (version 1.90) [29]. Analysis was restricted to variants (*n* = 1,165,557) with minor allele frequency (MAF) ≥ 0.01 and imputation quality score (INFO) ≥ 0.90. In Model 1, estimates were adjusted for sex and top 10 principal components (PCs). As described elsewhere [30–32], PCs were separately calculated by cohort among survivors of European-ancestry to adjust for the fine-scale population stratification. Model 2 estimates were additionally adjusted for clinical factors (including age at diagnosis, cancer type, surgery, chemotherapy, and radiotherapy as categorized in Supplementary Table 1) and demographic variables (including educational level, employment status, personal income, and marital status) as these factors may mediate (rather than confound) the association between genetic factors and PTSD. Missing values were coded as an additional category “unknown”. For variants with a *P*-value < 5 × 10^–6^ (marginal significant) in Model 1, associations were further assessed in the SJLIFE cohort followed by a meta-analysis using METAL [33]. *P*-value < 0.05 in the SJLIFE was considered as nominally significant. Variants with a *P*-value < 5 × 10^–8^, the most widely used threshold for common-variants GWAS [34], in the CCSS cohort or in meta-analysis were considered genome-wide significant.

To illustrate robustness of findings, associations of identified variants were examined by restricting PTSD cases to those with functional impairment or significant distress, respectively. To shed light on the specificity to PTSD, the associations of these variants were assessed by limiting to PTSD cases without clinically significant depression or anxiety, defined as a *t*-score ≥ 63 on BSI-18 Depression or Anxiety factors, respectively [35]. In addition, these variants were analyzed for the associations with overall and PTSD symptom subscores (i.e., re-experiencing, avoidance, and arousal symptoms) using linear regression.

Genetic markers were mapped using FUMA (Supplementary Methods) [36], followed by pathway analysis was performed using MAGMA (Supplementary Methods) [37]. We also estimated the heritability of PTSD based on the GWAS summary statistics using the LDSC (version 1.0.1) software package.

#### Comparison with other PTSD and psychosocial phenotypes

The associations of risk loci for PTSD identified in the CCS were compared to known risk loci for PTSD and stress-related disorders in other populations. Using the GWAS summary statistics of PTSD by the PTSD working group of PGC (PGC-PTSD) [11] and of stress-related disorders by the Lundbeck Foundation Initiative for Integrative Psychiatric Research (iPSYCH) [16], cross-study associations of lead SNPs of identified/known loci for PTSD/stress-related disorders were examined for heterogeneity [38]. Briefly, the ratio of the difference of estimates to its standard error was compared to the standard normal distribution to test the null hypothesis that the difference is zero. The regional associations of identified loci (500 kb on either side of the lead SNPs) were also visualized for these traits by integrating 1000 Genomes LD data with gene annotation tracks using LocusZoom (version 1.4) [39].

To shed light on the relations between genetic predispositions of other psychosocial traits and our phenotype, we calculated polygenic risk scores (PRSs) for these traits in CCS and analyzed the associations with PTSD risk (Supplementary Methods).

## Results

Of the CCSS participants [mean age 8.20 years at diagnosis, standard deviation (SD) 5.94; mean 31.87 years at time of PTSD assessment, SD 7.68], 671 (17.3%) met study criteria for PTSD on average 23.67 (SD 4.61) years after cancer diagnosis. Compared to individuals without PTSD, PTSD cases were more likely to have had radiotherapy [467 (69.6%) vs. 2058 (62.1%); Table 1]. Additionally, PTSD cases had lower socioeconomic status [high school or less: 140 (20.9%) vs. 500 (15.1%); were unemployed: 128 (19.1%) vs. 269 (8.1%); personal income below $20,000: 372 (55.4%) vs. 1344 (40.6%)], were more likely to be single [309 (46.1%) vs. 1,309 (39.5%)], smokers [137 (20.4%) vs. 362 (10.9%)], and physically inactive [278 (41.4%) vs. 1 165 (35.2%)]. Similar patterns were noted for SJLIFE participants [161 (11.0%) PTSD cases].

**Table 1:** Demographic and clinical characteristics of childhood cancer survivors in the CCSS and SJLIFE—mean ± SD or *N* (%).

		CCSS			SJLIFE	
	No PTSD	PTSD	*P*-value	No PTSD	PTSD	*P*-value
Individuals, *N*	3313	671	-	1306	161	-
Sex			0.067			0.112
Female	1753 (52.9)	381 (56.8)		595 (45.56)	84 (52.17)	
Male	1560 (47.1)	290 (43.2)		711 (54.44)	77 (47.83)	
At cancer diagnosis						
Age, year	8.14 ± 5.95	8.49 ± 5.87	0.165	8.56 ± 5.67	9.63 ± 6.02	0.025
Year of diagnosis			0.559			0.193
1960–1969	-	-		69 (5.28)	11 (6.83)	
1970–1975	820 (24.8)	158 (23.5)		47 (3.60)	8 (4.97)	
1976–1979	769 (23.2)	168 (25.0)		44 (3.37)	8 (4.97)	
1980–1989	1,724 (52.0)	345 (51.4)		306 (23.43)	47 (29.19)	
1990–1999	-	-		688 (52.68)	68 (42.24)	
2000–2004	-	-		152 (11.64)	19 (11.80)	
Cancer type			<0.001			0.004
Bone	286 (8.6)	68 (10.1)		88 (6.74)	13 (8.07)	
Central nervous system	378 (11.4)	114 (17.0)		212 (16.23)	13 (8.07)	
Hodgkin’s lymphoma	468 (14.1)	95 (14.2)		156 (11.94)	26 (16.15)	
Kidney	335 (10.1)	38 (5.7)		75 (5.74)	6 (3.73)	
Leukemia	1049 (31.7)	216 (32.2)		388 (29.71)	42 (26.09)	
Neuroblastoma	240 (7.2)	30 (4.5)		53 (4.06)	7 (4.35)	
Non-Hodgkin’s lymphoma	261 (7.9)	45 (6.7)		85 (6.51)	23 (14.29)	
Soft-tissue sarcoma	296 (8.9)	65 (9.7)		81 (6.20)	12 (7.45)	
Other	-	-		168 (12.86)	19 (11.80)	
Chemotherapy			0.698			0.013
No	688 (20.8)	134 (20.0)		272 (20.83)	22 (13.66)	
Alkylating agents	639 (19.3)	145 (21.6)		170 (13.02)	15 (9.32)	
Anthracyclines	285 (8.6)	52 (7.7)		225 (17.23)	24 (14.91)	
Both	925 (27.9)	182 (27.1)		521 (39.89)	87 (54.04)	
Other drugs	621 (18.7)	131 (19.5)		118 (9.04)	13 (8.07)	
Unknown	155 (4.7)	27 (4.0)		-	-	
Radiotherapy			<0.001			0.006
No	1126 (34.0)	187 (27.9)		646 (49.46)	77 (47.83)	
Radiation to brain	47 (1.4)	19 (2.8)		299 (22.89)	23 (14.29)	
Radiation but not to brain	1055 (31.8)	204 (30.4)		361 (27.64)	61 (37.89)	
Radiation, site unknown	956 (28.9)	244 (36.4)		-	-	
Unknown	129 (3.9)	17 (2.5)		-	-	
Surgery			0.456			0.996
No	663 (20.0)	145 (21.6)		81 (6.20)	10 (6.21)	
Yes	2489 (75.1)	499 (74.4)		1225 (93.80)	151 (93.79)	
Unknown	161 (4.9)	27 (4.0)		-	-	
At interview						
Age, year	31.82 ± 7.73	32.18 ± 7.44	0.252	29.37 ± 8.15	31.99 ± 7.65	<0.001
Education			<0.001			<0.001
High school or less	500 (15.1)	140 (20.9)		376 (28.79)	69 (42.86)	
Some college	1111 (33.5)	244 (36.4)		399 (30.55)	44 (27.33)	
College or more	1,702 (51.4)	287 (42.8)		434 (33.23)	32 (19.88)	
Unknown	-	-		97 (7.43)	16 (9.94)	
Employed			<0.001			<0.001
No	269 (8.1)	128 (19.1)		330 (25.27)	71 (44.10)	
Yes	3009 (90.8)	534 (79.6)		968 (74.12)	87 (54.04)	
Unknown	35 (1.1)	9 (1.3)		8 (0.61)	3 (1.86)	
Personal income			<0.001			0.018
Below $20,000	1344 (40.6)	372 (55.4)		699 (53.52)	104 (64.60)	
$20,000–39,999	951 (28.7)	141 (21.0)		292 (22.36)	35 (21.74)	
$40,000 or above	931 (28.1)	140 (20.9)		283 (21.67)	20 (12.42)	
Unknown	87 (2.6)	18 (2.7)		32 (2.45)	2 (1.24)	
Marital status			<0.001			<0.001
Single	1309 (39.5)	309 (46.1)		552 (42.27)	47 (29.19)	
Married or living as married	1,767 (53.3)	299 (44.6)		608 (46.55)	65 (40.37)	
Widowed, divorced, or separated	208 (6.3)	60 (8.9)		122 (9.34)	42 (26.09)	
Unknown	29 (0.9)	3 (0.4)		24 (1.84)	7 (4.35)	
Cigarette smoking			<0.001			<0.001
Never	2416 (72.9)	432 (64.4)		881 (67.41)	54 (33.54)	
Ever	532 (16.1)	102 (15.2)		177 (13.54)	26 (16.15)	
Current	362 (10.9)	137 (20.4)		242 (18.52)	78 (48.45)	
Unknown	3 (0.1)	0 (0)		7 (0.54)	3 (1.86)	
Physical activity			0.005			<0.001
Active	2115 (63.8)	384 (57.2)		752 (57.54)	64 (39.75)	
Inactive	1,165 (35.2)	278 (41.4)		547 (41.85)	97 (60.25)	
Unknown	33 (1.0)	9 (1.3)		8 (0.61)	0 (0.00)	
Health insurance			0.12			<0.001
Insured	2980 (89.9)	588 (87.6)		1033 (79.04)	106 (65.84)	
Uninsured	311 (9.4)	75 (11.2)		267 (20.43)	53 (32.92)	
Unknown	22 (0.7)	8 (1.2)		7 (0.54)	2 (1.24)	

*CCSS* Childhood Cancer Survivor Study, *CI* confidence interval, *N* number, *OR* odds ratio, *PTSD* posttraumatic stress disorder, *SD* standard deviation, *SJLIFE* St. Jude Lifetime Cohort Study.

### GWA analysis

**Fig. 1:**
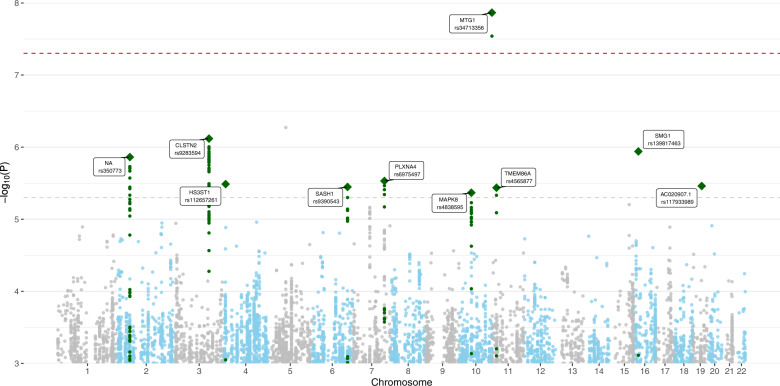
Manhattan plot from the GWAS of PTSD cases in the CCSS cohort, showing the top variants in 10 independent risk loci. CCSS Childhood Cancer Survivor Study, GWAS genome-wide association study, PTSD posttraumatic stress disorder. This analysis included 671 cases and 3313 controls. Single-nucleotide polymorphisms in green are in linkage disequilibrium (*r*^2^ < 0.1) with the index single-nucleotide polymorphisms (diamonds) and have a *P*-value < 0.001. Index variants located with a distance less than 400 kilobase are considered as 1 locus. The model was adjusted for sex and top 10 principal components. The point estimates are provided in Table 2 and Supplementary Table 2.

GWA analysis of PTSD cases in CCSS participants did not indicate genomic inflation (*λ* = 0.998; Supplementary Fig. 1). One locus, 10q26.3 was significantly associated with PTSD in CCS (rs34713356, Model 1: *P* = 1.36 × 10^–8^, OR 1.57, 95% CI 1.35–1.84), which was nominally significant in the SJLIFE cohort (*P* = 0.047, OR 1.37, 95% CI 1.00–1.87; Fig. 1 and Table 2). Nine loci reached marginal significance in the CCSS cohort; of these, the lead SNP in locus 6q24.3-q25.1 (rs9390543, *P* = 3.56 × 10^–6^, OR 0.75, 95% CI 0.67–0.85) was genome-wide significant in a meta-analysis with the SJLIFE cohort (*P* = 2.02 × 10^–8^, OR 0.75, 95% CI 0.68–0.83; Table 2). Additional adjustment for clinical and socioeconomic characteristics yielded similar associations for both variants (Model 2 in Table 1 and Supplementary Fig. 2). Risk of PTSD by genotype indicated an additive association for both top variants (Supplementary Table 1). Loci which were not replicated or did not reach genome-wide significance in meta-analysis are reported in Supplementary Table 2. The SNP-based heritability of PTSD was estimated at 0.024 (SD 0.109, *P* > 0.05) in the CCSS sample.

**Table 2:** Lead SNPs in top loci associated with PTSD cases in the discovery cohort (CCSS) and their results in the replication cohort (SJLIFE) and in meta-analysis^a^.

								Model 1^b^		Model 2^c^	
Chr	Position	SNP	Gene	A1	A2	Sample	RAF	OR (95% CI)	*P*	OR (95% CI)	*P*
10	135208461	rs34713356	*MTG1*	A	G	CCSS	0.14	1.57 (1.35–1.84)	1.36 × 10^–8^	1.61 (1.37–1.90)	6.41 × 10^–9^
						SJLIFE	0.15	1.37 (1.00–1.87)	0.047	1.39 (0.99–1.95)	0.058
						Meta-analysis	-	1.53 (1.34–1.75)	8.55 × 10^–10^	1.57 (1.36–1.82)	1.38 × 10^–9^
6	148472854	rs9390543	*SASH1*	G	A	CCSS	0.45	0.75 (0.67–0.85)	3.56 × 10^–6^	0.74 (0.65–0.84)	1.86 × 10^–6^
						SJLIFE	0.43	0.74 (0.58–0.93)	0.011	0.74 (0.58–0.96)	0.02
						Meta-analysis	-	0.75 (0.68–0.83)	2.02 × 10^–8^	0.74 (0.66–0.83)	1.18 × 10^–7^

*A1* Risk allele, *A2* reference allele, *CCSS* Childhood Cancer Survivor Study, *Chr* chromosome, *CI* confidence interval, *OR* odds ratio, *PTSD* posttraumatic stress disorder, *RAF* risk allele frequency, *SJLIFE* St. Jude Lifetime Study, *SNP* single-nucleotide polymorphism.

^a^Index variants are linkage disequilibrium independent (*r*^2^ < 0.1) and are merged into 1 locus when located with a distance less than 400 kilobases. Genes were mapped in either positional (i.e., SNPs physically located inside a gene with up to 200 kilobase windows) or eQTL mapping (based on brain and blood samples from GTEx project as described previously).

^b^Estimates were adjusted for sex and top 10 principal components.

^c^Estimates were additional adjusted for age at cancer diagnosis, cancer type, surgery, chemotherapy, radiotherapy, educational level, employment status, personal income, and marital status.

In sensitivity analyses, the associations of rs34713356 and rs9390543 remained comparable after restricting analysis to PTSD without significant depression or anxiety and to PTSD with functional impairment or significant distress (Supplementary Table 3). In addition, effect sizes of associations with the overall PTSD symptom score and subscores were highly consistent for both variants, although associations of rs9390543 with arousal and avoidance symptom scores were not significant at a nominal level.

Examining total PTSD symptom scores as a secondary outcome demonstrated a significant association with one locus in DOK7 (rs573108942, *P* = 2.45 × 10^–8^) in the CCSS cohort, while 14 loci reached marginal significance level (Supplementary Fig. 3 and Supplementary Table 4). However, none were replicated in the SJLIFE cohort nor significant in meta-analysis.

### Comparison with other PTSD and psychosocial phenotypes

Lead SNPs rs34713356 and rs9390543 were not associated with PTSD in the PGC-PTSD GWAS or with stress-related disorders in iPSYCH GWAS (*P* for heterogeneity <5 × 10^−6^; Table 3). The patterns of associations within both loci were different from the regional associations in PGC-PTSD or iPSYCH GWAS (Supplementary Figs. 4 and 5). However, similar associations in the CCSS cohort were noted for genetic variants previously identified by PGC-PTSD and iPSYCH, respectively (effect sizes were similar and P for heterogeneity >0.05).

**Table 3:** The associations of lead SNPs with PTSD in childhood cancer survivors (CCSS), PTSD in general population (PGC), and stress-related disorders (iPSYCH).

					CCSS		PGC			iPSYCH		
Chr	Position	SNP	A1	A2	OR (95% CI)	*P*	OR (95% CI)	*P*	*P*_het_	OR (95% CI)	*P*	*P*_het_
Lead SNPs associated with PTSD in cancer survivors, CCSS												
10	135208461	rs34713356	A	G	1.57 (1.35–1.84)	1.36 × 10^–8^	1.03 (0.96–1.11)	0.382	1.96 × 10^–6^	- ^a^	**-**	**-**
6	148472854	rs9390543	G	A	0.75 (0.67–0.85)	3.56 × 10^–6^	1.02 (0.99–1.05)	0.173	1.40 × 10^–6^	1.02 (0.98–1.06)	0.389	2.82 × 10^–6^
Lead SNPs associated with PTSD in general population, PGC (PMID: 31594949)												
6	157789333	rs34517852	A	T	1.16 (1.02–1.31)	0.019	1.12 (1.08–1.16)	3.16 × 10^–9^	0.584	1.03 (0.99–1.07)	0.158	0.0781
6	162163506	rs9364611	T	C	0.93 (0.77–1.12)	0.435	0.88 (0.85–0.92)	4.36 × 10^–8^	0.601	0.99 (0.93–1.04)	0.664	0.533
Lead SNPs associated with stress-related disorders, iPSYCH (PMID: 31116379)												
1	66407352	rs7528604	A	G	0.89 (0.79–1.00)	0.058	0.99 (0.96–1.02)	0.392	0.102	0.89 (0.86–0.93)	5.71 × 10^–9^	0.951

*A1* Risk allele, *A2* reference allele, *CCSS* Childhood Cancer Survivor Study, *Chr* chromosome, *CI* confidence interval, *iPSYCH* The Lundbeck Foundation Initiative for Integrative Psychiatric Research, *OR* odds ratio, PGC Psychiatric Genomics Consortium, *P*_het_ *P* for heterogeneity by comparing the OR with that in CCSS, *PTSD* posttraumatic stress disorder, *SNP* single-nucleotide polymorphism.

^a^The association of variant rs34713356 is not available in iPSYCH study.

**Fig. 2:**
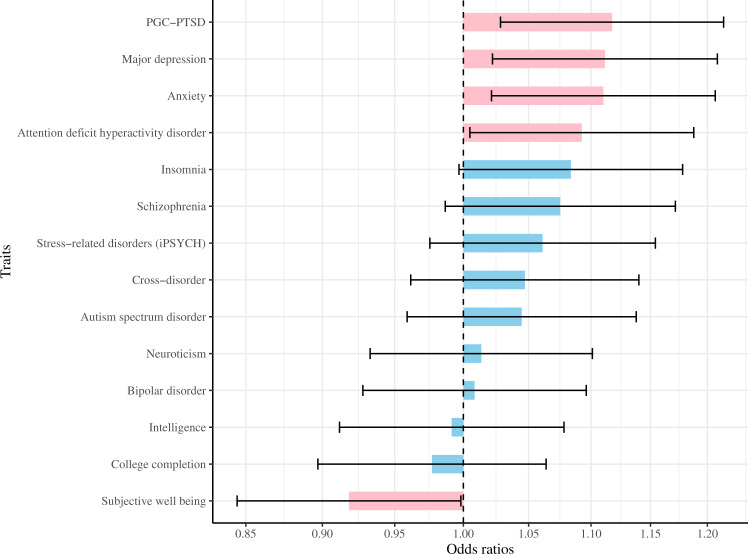
Polygenic associations between common psychosocial traits and PTSD in childhood cancer survivors. *iPSYCH* The Lundbeck Foundation Initiative for Integrative Psychiatric Research, *PGC* Psychiatric Genomics Consortium, *PTSD* posttraumatic stress disorder. We performed polygenic risk score (PRS) analyses using genetic variants of *P*-value < 0.005 from corresponding GWASs of psychosocial traits. Odds ratios (per 1 standard deviation of standardized PRS) of PTSD in cancer survivors are shown as *x*-axis, while error bars indicate 95% confidence intervals. Pink bars denote significant associations (*P*-value < 0.05).

PTSD risk in CCS was associated with the PRS for PGC-PTSD GWAS (OR 1.12 per *z*-score, 95% CI 1.03–1.21, *P* = 0.009; Fig. 2) but not with the PRS for iPSYCH GWAS (OR 1.06, 95% CI 0.98–1.15, *P* = 0.17). When examining the relations between genetic markers of a range of psychosocial traits and PTSD, PRS for major depression, anxiety, and attention deficit hyperactivity disorder were associated with increased risk of PTSD in survivors, whereas PRS for subjective well-being was inversely associated with PTSD in survivors (nominal *P* < 0.05; Fig. 2).

### Functional annotation

Locus 10q26.3 was positionally mapped to *MTG1* and the anchored lead SNP rs34713356 is an intron variant. However, locus 10q26.3 was mapped to *ECHS1* via eQTL, which shows that the allele A of rs34713356 was associated with decreased expression of *ECHS1* in blood samples (adjusted *P* = 1.20 × 10^–3^). Locus 6q24.3-q25.1 is an intergenic locus and mapped to *SASH1*. The allele G of rs9390543 was associated with increased expression of *SASH1* in blood samples (adjusted *P* = 0.003).

In the pathway analysis, 16 molecular pathways were associated with PTSD in survivors after multiple-comparison correction (adjusted *P* < 0.05; Supplementary Table 5). The top three enriched pathways included pathways regulating the ARF family proteins associated with GTP-bound active state, modulating cytokine produced by T-helper 1 cells, and governing programmed necrotic cell death.

## Discussion

To our knowledge, this is the first study to describe the genomic characterization of PTSD after childhood cancer. Importantly, we identified two risk loci for PTSD with common-variants that were replicable in an independent cohort of CCSS and significant in genome-wide meta-analysis. Moreover, these two loci associated with PTSD in CCS have not been linked to PTSD or stress-related disorders in studies of exposures to interpersonal or combat trauma.

In the present study, we identified two genetic loci not previously associated with PTSD. *ECHS1* associated with locus 10q26.3 is a key enzyme involved in mitochondrial fatty acid β-oxidation and many metabolic pathways through catalyzing the hydration of enoyl-coenzyme A [40]. Deficiency of *ECHS1* protein disrupts mitochondrial functions and may lead to brain pathology, such as Leigh syndrome characterized by psychomotor regression [41], and has been linked to psychiatric symptoms [42, 43]. Indeed, mitochondrial dysfunction may lead to PTSD symptomatology through abnormal fear learning, brain network activation, steroidogenesis, and inflammation [44]. *SASH1* in locus 6q24.3-q25.1 encodes a scaffold protein, which stimulates cytokine production through NF-κB signaling pathway and facilitate endothelial responses to inflammation/infection [45]. This is in line with the well-documented association between PTSD and pro-inflammatory state and immune imbalance [46]. Of note, NF-κB signaling and the role in inflammation have been linked to some cancer [47]. It is possible that this finding is explained by the differential distribution, if any, of cancer types between PTSD cases and controls. However, our analysis with additional control for cancer type (Model 2) has yielded very similar results. In addition, neither loci (*r*^2^ > 0.1 and 500KB on either side of the lead SNP) have been associated with cancer according to the LDlink online tool (https://ldlink.nci.nih.gov/) [48]. It has also been suggested that genetic variants of *SASH*1 are associated with other psychiatric disorders and traits, e.g., comorbid major depression, alcohol dependence [49], and smoking behavior [50]. In addition, our pathway analysis lends further support to the involvement of energy production process and immune regulation pathways. Although we cannot completely rule out the possibility of chance findings, it is plausible that both loci are particularly relevant to PTSD after childhood cancer experiences. In contrast, these loci have a null association in PGC-PTSD or iPSYCH [11, 16] and the signal patterns were very different from our results. The different results might be explained by the different phenotypes—predominately interpersonal violence-associated PTSD in PGC-PTSD, stress-related disorders in iPSYCH, and cancer-associated PTSD in our sample. Future research is needed to understand the genetic basis for PTSD and related disorders that develop after different traumatic exposures (e.g., interpersonal trauma, life-threatening disease, and natural disaster).

In addition to identifying genetic loci specifically associated with PTSD in CCS, results lend support to a substantial genetic overlap with PTSD developed after other traumatic events. For example, known genetic loci identified by PGC-PTSD and iPSYCH were found to have largely similar point estimates in our sample (e.g., rs9364611, OR = 0.88 in PGC-PTSD and 0.93 in CCSS), though the associations in our sample were not statistically significant. Moreover, analysis showing a positive association between PRS for PGC-PTSD and PTSD risk among CCS supports the shared genetic architecture. If replicated in future studies, this may provide feasible means to develop novel nomograms to predict PTSD risk in adulthood among CCS. However, a weaker, non-significant association was also observed between PRS for stress-related disorders (iPSYCH) and PTSD in our cohort, likely because of the heterogeneity between PTSD and acute stress reaction/adjustment disorder, all included in the iPSYCH study. Additionally, PTSD risk in CCS to be positively associated with PRS for depression, anxiety, and ADHD, whereas inversely associated with PRS for subjective well-being. This is in line with reports on the genetic correlations between PTSD and other traits [11]. The common genetic origin may highlight the potential burden of PTSD comorbid with other psychiatric disorders, which has been well-documented in population settings [51–53], but not recognized in CCS until recently [54, 55].

In addition, our pathway analysis suggests that regulation of programmed necrotic cell death may play a role in the PTSD development among CCS. Programmed cell death is an essential mechanism to regulate number and function of neurons in adult brain [56]. Animal studies have shown that traumatic exposure and chronic stress increase the apoptotic cell death in hippocampus and cerebral cortex, respectively [57, 58]. Moreover, a higher rate of neuronal apoptosis has been found in the hippocampus of PTSD-like animal model following severe traumatic stress [59]. It is plausible that the genetic liability to programmed necrotic apoptosis in brain interacts with the neurotoxicity induced by cancer treatment [60]. Indeed, CCS who received cranial radiation before age 4 have doubled risk of developing PTSD compared to their siblings [4]. However, future studies are needed to understand the potential mechanism of neuronal apoptosis underlying PTSD.

Several limitations should be considered. First, we aimed to capture survivors with symptoms persisting from childhood into adulthood or new onset symptoms in adulthood. We may have missed PTSD symptoms that developed immediately following the cancer but resolved over time. However, misclassifying these “resolved” cases into controls would have led to attenuated associations; and our results should only be interpreted as risk loci for PTSD symptoms present >20 years after the traumatic exposure. Second, we identified potential PTSD cases based on self-report checklist, which is not equivalent to clinical diagnosis. Moreover, we used PTSD diagnostic criteria based on DSM-IV; some PTSD cases may not meet current PTSD criteria as they were revised for DSM-5 [61]. However, the consistent associations across PTSD symptom subscores (i.e., re-experiencing, avoidance, arousal) may help alleviate such concern. In addition, the importance of capturing subthreshold PTSD is widely recognized, as it identifies individuals with a significant symptom burden and impaired functional outcomes and quality of life [62–64]. Of note, similar associations were observed after restricting to cases showing functional impairment or significant distress, supporting the validity of PTSD ascertainment. Third, the reference group in CCSS consisted of survivors who did not meet PTSD diagnostic criteria in young adulthood. Some may manifest milder PTSD symptoms, have previously had PTSD but recovered, or have PTSD related to other traumatic events (not measured in the survey), which may have led to attenuated associations. Furthermore, assessment of PTSD in the SJLIFE cohort captured symptoms relevant to all traumatic events, while in the CCSS cohort PTSD symptoms were specifically assessed in relation to the childhood cancer experience. PTSD symptoms related to other traumatic exposures were not assessed in the CCSS cohort. This could limit replication of results, as the prevalence of PTSD in the two samples were statistically different (17% in the CCSS cohort vs. 11% in the SJLIFE cohort). However, the advantage of using these two cohorts is that through the replication and meta-analysis, the two discovered loci are specifically associated with cancer-related PTSD in the CCSS and stand out from the heterogenous traumatic exposures experienced by all survivors in the SJLIFE. Last, we excluded participants of non-European-ancestry due to the relatively small sample size to reach a genome-wide significance. Future studies including diverse racial and ethnic backgrounds are needed.

In conclusion, our findings on CCS support the role of genetic variation in the development of PTSD. If confirmed in independent populations, the identified common-variants may help develop risk stratification for early detection and intervention among CCS. Future studies based on these novel findings are needed to understand the heterogeneity of biology underlying PTSD developed after different traumatic events, and to develop interventions that address these vulnerabilities.

## Supplementary information

Supplementary methods, figures and tables

## References

[1] Jo Bush NG, Linda M. Psychosocial nursing care along the cancer continuum. 3rd ed. Pittsburgh, PA: Oncology Nursing Society; 2018.

[2] Stark DP, House A (2000). Anxiety in cancer patients. Br J Cancer.

[3] Brinkman TM, Recklitis CJ, Michel G, Grootenhuis MA, Klosky JL (2018). Psychological symptoms, social outcomes, socioeconomic attainment, and health behaviors among survivors of childhood cancer: current state of the literature. J Clin Oncol.

[4] Stuber ML, Meeske KA, Krull KR, Leisenring W, Stratton K, Kazak AE (2010). Prevalence and predictors of posttraumatic stress disorder in adult survivors of childhood cancer. Pediatrics..

[5] Michel G, François C, Harju E, Dehler S, Roser K (2019). The long-term impact of cancer: evaluating psychological distress in adolescent and young adult cancer survivors in Switzerland. Psychooncology.

[6] Zeltzer LK, Recklitis C, Buchbinder D, Zebrack B, Casillas J, Tsao JC (2009). Psychological status in childhood cancer survivors: a report from the Childhood Cancer Survivor Study. J Clin Oncol.

[7] Allen J, Willard VW, Klosky JL, Li C, Srivastava DK, Robison LL (2018). Posttraumatic stress-related psychological functioning in adult survivors of childhood cancer. J Cancer Surviv.

[8] Duncan LE, Cooper BN, Shen H (2018). Robust findings from 25 years of PSTD genetics research. Curr Psychiatry Rep.

[9] Sartor CE, Grant JD, Lynskey MT, McCutcheon VV, Waldron M, Statham DJ (2012). Common heritable contributions to low-risk trauma, high-risk trauma, posttraumatic stress disorder, and major depression. Arch Gen Psychiatry.

[10] Stein MB, Jang KL, Taylor S, Vernon PA, Livesley WJ (2002). Genetic and environmental influences on trauma exposure and posttraumatic stress disorder symptoms: a twin study. Am J Psychiatry.

[11] Nievergelt CM, Maihofer AX, Klengel T, Atkinson EG, Chen CY, Choi KW (2019). International meta-analysis of PTSD genome-wide association studies identifies sex- and ancestry-specific genetic risk loci. Nat Commun.

[12] Stein MB, Chen CY, Ursano RJ, Cai T, Gelernter J, Heeringa SG (2016). Genome-wide association studies of posttraumatic stress disorder in 2 cohorts of US army soldiers. JAMA Psychiatry.

[13] Stein MB, Levey DF, Cheng Z, Wendt FR, Harrington K, Pathak GA (2021). Genome-wide association analyses of post-traumatic stress disorder and its symptom subdomains in the Million Veteran Program. Nat Genet.

[14] Dalvie S, Maihofer AX, Coleman JRI, Bradley B, Breen G, Brick LA (2020). Genomic influences on self-reported childhood maltreatment. Transl Psychiatry.

[15] Ratanatharathorn A, Koenen KC, Chibnik LB, Weisskopf MG, Rich-Edwards JW, Roberts AL (2021). Polygenic risk for autism, attention-deficit hyperactivity disorder, schizophrenia, major depressive disorder, and neuroticism is associated with the experience of childhood abuse. Mol Psychiatry.

[16] Meier SM, Trontti K, Purves KL, Als TD, Grove J, Laine M (2019). Genetic variants associated with anxiety and stress-related disorders: a Genome-Wide Association Study and Mouse-Model Study. JAMA Psychiatry.

[17] Akhavanfard S, Padmanabhan R, Yehia L, Cheng F, Eng C (2020). Comprehensive germline genomic profiles of children, adolescents and young adults with solid tumors. Nat Commun.

[18] Zhang J, Walsh MF, Wu G, Edmonson MN, Gruber TA, Easton J (2015). Germline mutations in predisposition genes in pediatric cancer. N. Engl J Med.

[19] Robison LL, Armstrong GT, Boice JD, Chow EJ, Davies SM, Donaldson SS (2009). The Childhood Cancer Survivor Study: a National Cancer Institute-supported resource for outcome and intervention research. J Clin Oncol.

[20] Leisenring WM, Mertens AC, Armstrong GT, Stovall MA, Neglia JP, Lanctot JQ (2009). Pediatric cancer survivorship research: experience of the Childhood Cancer Survivor Study. J Clin Oncol.

[21] Morton LM, Sampson JN, Armstrong GT, Chen TH, Hudson MM, Karlins E (2017). Genome-wide association study to identify susceptibility loci that modify radiation-related risk for breast cancer after childhood cancer. J Natl Cancer Inst.

[22] Howell CR, Bjornard KL, Ness KK, Alberts N, Armstrong GT, Bhakta N (2021). Cohort profile: the St. Jude Lifetime Cohort Study (SJLIFE) for paediatric cancer survivors. Int J Epidemiol.

[23] Foa EB, Cashman L, Jaycox L, Perry K (1997). The validation of a self-report measure of posttraumatic stress disorder: The Posttraumatic Diagnostic Scale. Psychological Assess.

[24] Zeltzer LK, Lu Q, Leisenring W, Tsao JC, Recklitis C, Armstrong G (2008). Psychosocial outcomes and health-related quality of life in adult childhood cancer survivors: a report from the childhood cancer survivor study. Cancer Epidemiol Biomark Prev.

[25] Blanchard EB, Jones-Alexander J, Buckley TC, Forneris CA (1996). Psychometric properties of the PTSD Checklist (PCL). Behav Res Ther.

[26] Smith MY, Redd W, DuHamel K, Vickberg SJ, Ricketts P (1999). Validation of the PTSD Checklist-Civilian Version in survivors of bone marrow transplantation. J Trauma Stress.

[27] McHorney CA, Ware JE, Raczek AE (1993). The MOS 36-Item Short-Form Health Survey (SF-36): II. Psychometric and clinical tests of validity in measuring physical and mental health constructs. Med Care.

[28] Derogatis LR. BSI 18, Brief Symptom Inventory 18: administration, scoring and procedures manual. Minneapolis, MN: NCS Pearson, Inc.; 2001.

[29] Chang CC, Chow CC, Tellier LC, Vattikuti S, Purcell SM, Lee JJ (2015). Second-generation PLINK: rising to the challenge of larger and richer datasets. Gigascience..

[30] Sapkota Y, Cheung YT, Moon W, Shelton K, Wilson CL, Wang Z (2019). Whole-genome sequencing of childhood cancer survivors treated with cranial radiation therapy identifies 5p15.33 locus for stroke: a report from the St. Jude Lifetime Cohort Study. Clin Cancer Res.

[31] Sapkota Y, Qin N, Ehrhardt MJ, Wang Z, Chen Y, Wilson CL (2021). Genetic variants associated with therapy-related cardiomyopathy among childhood cancer survivors of African Ancestry. Cancer Res.

[32] Sapkota Y, Wilson CL, Zaidi AK, Moon W, Fon Tacer K, Lu L (2020). A novel locus predicts spermatogenic recovery among childhood cancer survivors exposed to alkylating agents. Cancer Res.

[33] Willer CJ, Li Y, Abecasis GR (2010). METAL: fast and efficient meta-analysis of genomewide association scans. Bioinformatics.

[34] Risch N, Merikangas K (1996). The future of genetic studies of complex human diseases. Science..

[35] Recklitis CJ, Blackmon JE, Chang G (2017). Validity of the Brief Symptom Inventory-18 (BSI-18) for identifying depression and anxiety in young adult cancer survivors: comparison with a structured clinical diagnostic interview. Psychol Assess.

[36] Watanabe K, Taskesen E, van Bochoven A, Posthuma D (2017). Functional mapping and annotation of genetic associations with FUMA. Nat Commun.

[37] de Leeuw CA, Mooij JM, Heskes T, Posthuma D (2015). MAGMA: generalized gene-set analysis of GWAS data. PLoS Comput Biol.

[38] Altman DG, Bland JM (2003). Interaction revisited: the difference between two estimates. BMJ..

[39] Pruim RJ, Welch RP, Sanna S, Teslovich TM, Chines PS, Gliedt TP (2010). LocusZoom: regional visualization of genome-wide association scan results. Bioinformatics..

[40] Burgin HJ, McKenzie M (2020). Understanding the role of OXPHOS dysfunction in the pathogenesis of ECHS1 deficiency. FEBS Lett.

[41] Peters H, Buck N, Wanders R, Ruiter J, Waterham H, Koster J (2014). ECHS1 mutations in Leigh disease: a new inborn error of metabolism affecting valine metabolism. Brain..

[42] Satogami K, Takahashi S, Kose A, Shinosaki K (2017). Schizophrenia-like symptoms in a patient with Leigh syndrome. Asian J Psychiatr.

[43] Mnif L, Sellami R, Masmoudi J (2015). Schizophrenia and Leigh syndrome, a simple comorbidity or the same etiopathogeny: about a case. Pan Afr Med J..

[44] Preston G, Kirdar F, Kozicz T (2018). The role of suboptimal mitochondrial function in vulnerability to post-traumatic stress disorder. J Inherit Metab Dis.

[45] Dauphinee SM, Clayton A, Hussainkhel A, Yang C, Park YJ, Fuller ME (2013). SASH1 is a scaffold molecule in endothelial TLR4 signaling. J Immunol.

[46] Passos IC, Vasconcelos-Moreno MP, Costa LG, Kunz M, Brietzke E, Quevedo J (2015). Inflammatory markers in post-traumatic stress disorder: a systematic review, meta-analysis, and meta-regression. Lancet Psychiatry.

[47] Hoesel B, Schmid JA (2013). The complexity of NF-kappaB signaling in inflammation and cancer. Mol Cancer.

[48] Lin SH, Brown DW, Machiela MJ (2020). LDtrait: an online tool for identifying published phenotype associations in linkage disequilibrium. Cancer Res.

[49] Zhou H, Polimanti R, Yang BZ, Wang Q, Han S, Sherva R (2017). Genetic risk variants associated with comorbid alcohol dependence and major depression. JAMA Psychiatry.

[50] Argos M, Tong L, Pierce BL, Rakibuz-Zaman M, Ahmed A, Islam T (2014). Genome-wide association study of smoking behaviours among Bangladeshi adults. J Med Genet.

[51] Brady KT, Killeen TK, Brewerton T, Lucerini S (2000). Comorbidity of psychiatric disorders and posttraumatic stress disorder. J Clin Psychiatry.

[52] Rytwinski NK, Scur MD, Feeny NC, Youngstrom EA (2013). The co-occurrence of major depressive disorder among individuals with posttraumatic stress disorder: a meta-analysis. J Trauma Stress.

[53] Biederman J, Petty CR, Spencer TJ, Woodworth KY, Bhide P, Zhu J (2013). Examining the nature of the comorbidity between pediatric attention deficit/hyperactivity disorder and post-traumatic stress disorder. Acta Psychiatr Scand.

[54] D’Agostino NM, Edelstein K, Zhang N, Recklitis CJ, Brinkman TM, Srivastava D (2016). Comorbid symptoms of emotional distress in adult survivors of childhood cancer. Cancer.

[55] Brinkman TM, Li C, Vannatta K, Marchak JG, Lai JS, Prasad PK (2016). Behavioral, social, and emotional symptom comorbidities and profiles in adolescent survivors of childhood cancer: a report from the Childhood Cancer Survivor Study. J Clin Oncol.

[56] Duman RS (2009). Neuronal damage and protection in the pathophysiology and treatment of psychiatric illness: stress and depression. Dialogues Clin Neurosci.

[57] Lee J, Duan W, Mattson MP (2002). Evidence that brain-derived neurotrophic factor is required for basal neurogenesis and mediates, in part, the enhancement of neurogenesis by dietary restriction in the hippocampus of adult mice. J Neurochem.

[58] Bachis A, Mallei A, Cruz MI, Wellstein A, Mocchetti I (2008). Chronic antidepressant treatments increase basic fibroblast growth factor and fibroblast growth factor-binding protein in neurons. Neuropharmacology..

[59] Gao J, Wang H, Liu Y, Li YY, Chen C, Liu LM (2014). Glutamate and GABA imbalance promotes neuronal apoptosis in hippocampus after stress. Med Sci Monit.

[60] Alessi I, Caroleo AM, de Palma L, Mastronuzzi A, Pro S, Colafati GS (2022). Short and long-term toxicity in pediatric cancer treatment: central nervous system damage. Cancers (Basel).

[61] Kilpatrick DG, Resnick HS, Milanak ME, Miller MW, Keyes KM, Friedman MJ (2013). National estimates of exposure to traumatic events and PTSD prevalence using DSM-IV and DSM-5 criteria. J Trauma Stress.

[62] El-Gabalawy R, Blaney C, Tsai J, Sumner JA, Pietrzak RH (2018). Physical health conditions associated with full and subthreshold PTSD in U.S. military veterans: results from the National Health and Resilience in Veterans Study. J Affect Disord.

[63] Chen C, Salim R, Rodriguez J, Singh R, Schechter C, Dasaro CR (2020). The burden of subthreshold posttraumatic stress disorder in World Trade Center responders in the second decade after 9/11. J Clin Psychiatry.

[64] Brancu M, Mann-Wrobel M, Beckham JC, Wagner HR, Elliott A, Robbins AT (2016). Subthreshold posttraumatic stress disorder: a meta-analytic review of DSM-IV prevalence and a proposed DSM-5 approach to measurement. Psychol Trauma..

