# Supplementary material for: Genome-wide association study of posttraumatic stress disorder among childhood cancer survivors: results from the Childhood Cancer Survivor Study and the St. Jude Lifetime Cohort
Source: Transl Psychiatry. 2022 Aug 23;12:342. doi: 10.1038/s41398-022-02110-w (PMC9399128; doi:10.1038/s41398-022-02110-w)
Supplement: Supplementary file 1 — Supplementary methods, figures and tables [file 41398_2022_2110_MOESM1_ESM.docx]

**Supplementary Information**

Supplement to: Lu D, Sapkota Y, Valdimarsdóttir UA et al. Genome-wide association study of posttraumatic stress disorder among childhood cancer survivors: results from the Childhood Cancer Survivor Study and the St. Jude Lifetime Cohort.

**Supplementary Methods**

*Genotyping, imputation and quality control*

In the CCSS cohort, DNA was extracted using standard methods from blood, saliva (Oragene), or buccal cells (collected from mouthwash). Whole-genome amplification was performed for samples without sufficient DNA. Genotyping was conducted at the Cancer Genomics Research Laboratory of the National Cancer Institute on the Illumina HumanOmni5Exome array (San Diego, CA). Ancestry was estimated using the Genotyping Library and Utilities struct.admix module with HapMap data as the reference population. Imputation was performed based on the 1000 Genomes Project phase 3 release using IMPUTE version 2.3.0, resulting in genotypes for a total of 26,135,905 single nucleotide polymorphisms (SNPs) and small insertions or deletions. The genotype data have been deposited at dbGaP (accession number: phs001327.v1.p1).

In the SJLIFE participants, DNA from blood samples was isolated using either the QIAamp DNA Blood Mini Kit (Qiagen cat#51106) or DNeasy Blood & Tissue Kit (cat# 69506). As previously described,[1] whole genome sequencing was performed at the HudsonAlpha Institute for Biotechnology Genomic Services Laboratory (Huntsville, Alabama) using the Illumina HiSeq X10 sequencers to generate 360 million paired-ends reads, each 150 base pairs (bp) in length, for each sample (30x average coverage). After quality control, information on approximately 84.3 million autosomal single nucleotide variants and small insertions and deletions (indels) was available for analysis. As described elsewhere,[1-3] ancestry for SJLIFE participants was determined based on the principal component analysis with 1000 Genomes as the reference population. Briefly, we combined the SJLIFE genotype data of an independent set of common variants with 26 global populations from the 1000 Genomes Project and performed EIGENSTRAT-based Principal Component Analysis (PCA) to obtain the first two principal components (PC1 and PC2), implemented in the PLINK 1·90b software. The mean PC1 and PC2 scores of the 1000 Genomes European populations (CEU- Utah Residents (CEPH) with Northern and Western European Ancestry; TSI - Toscani in Italia; FIN - Finnish in Finland; GBR - British in England and Scotland; and IBS-Iberian Population in Spain) were used as a reference and any SJLIFE sample within three standard deviations from these along PC1 or PC2 were classified as an European to be included for this study. Regarding relatedness, we used estimates of pairwise identity-by-descent based on the genotype data of an independent set of common variants to detect pairs of survivors who appeared too similar to each other. From each cryptically related pair (defined based as PI_HAT of >0.25), we retained the survivor with a lower missingness.[1-3]

*Polygenic risk score analysis*

We used published GWAS summary statistics of European ancestry (whenever possible), including GWASs of PGC-PTSD[4], iPSYCH PTSD[5], major depression[6], anxiety[7], attention deficit/hyperactivity disorder[8], insomnia[9], schizophrenia[10], cross-disorder[11], autism spectrum disorder[12], neuroticism[13], bipolar disorder[14], intelligence[15], college completion[16], and subjective well being[17]. CCSS were not part of above GWASs and sample overlap should be less likely. We filtered SNPs with MAF ≥ 0.01 and INFO ≥ 0.90 (whenever possible) and restricted to overlapped SNPs with our genotype data. LD clumping (*r*^2^ < 0.1 in 1000kb window) was performed in PLINK using the 1000 Genomes Project European samples as LD reference (after removing the HLA region). PRS were calculated for each trait in the CCSS sample as the weighted sum of the imputed SNP alleles with a P value < 0.005 weighted by the allele effect size from the published GWASs. We then standardized scores for each trait and examined the associations with PTSD using logistic regression with adjustment for sex, age at diagnosis, and top 10 principal components in R (version 3.6.1).

*Functional mapping and annotation*

FUMA,[18] a web-based platform ([http://fuma.ctglab.nl/](about:blank)), was used to perform mapping and annotation. SNPs were mapped in either positional (i.e., SNPs physically located inside a gene with up to 10 kb windows set as the default in FUMA)) or cis-expression Quantitative Trait Loci (eQTL) mapping (based on blood samples from the eQTLGen [https://www.eqtlgen.org/index.html](about:blank)).[19] Pathway analysis was conducted to further annotate findings using MAGMA.[20] The P values in eQTL and pathway analyses were corrected for multiple comparison using the false discovery rate method.[21]

*References*

1. Sapkota Y, Cheung YT, Moon W, Shelton K, Wilson CL, Wang Z, et al. Whole-Genome Sequencing of Childhood Cancer Survivors Treated with Cranial Radiation Therapy Identifies 5p15.33 Locus for Stroke: A Report from the St. Jude Lifetime Cohort Study. Clin Cancer Res. 2019;25(22):6700-8.

2. Sapkota Y, Qin N, Ehrhardt MJ, Wang Z, Chen Y, Wilson CL, et al. Genetic Variants Associated with Therapy-Related Cardiomyopathy among Childhood Cancer Survivors of African Ancestry. Cancer Res. 2021;81(9):2556-65.

3. Sapkota Y, Wilson CL, Zaidi AK, Moon W, Fon Tacer K, Lu L, et al. A Novel Locus Predicts Spermatogenic Recovery among Childhood Cancer Survivors Exposed to Alkylating Agents. Cancer Res. 2020;80(17):3755-64.

4. Nievergelt CM, Maihofer AX, Klengel T, Atkinson EG, Chen CY, Choi KW, et al. International meta-analysis of PTSD genome-wide association studies identifies sex- and ancestry-specific genetic risk loci. Nat Commun. 2019;10(1):4558.

5. Meier SM, Trontti K, Purves KL, Als TD, Grove J, Laine M, et al. Genetic Variants Associated With Anxiety and Stress-Related Disorders: A Genome-Wide Association Study and Mouse-Model Study. JAMA Psychiatry. 2019.

6. Wray NR, Ripke S, Mattheisen M, Trzaskowski M, Byrne EM, Abdellaoui A, et al. Genome-wide association analyses identify 44 risk variants and refine the genetic architecture of major depression. Nat Genet. 2018;50(5):668-81.

7. Otowa T, Hek K, Lee M, Byrne EM, Mirza SS, Nivard MG, et al. Meta-analysis of genome-wide association studies of anxiety disorders. Mol Psychiatry. 2016;21(10):1391-9.

8. Demontis D, Walters RK, Martin J, Mattheisen M, Als TD, Agerbo E, et al. Discovery of the first genome-wide significant risk loci for attention deficit/hyperactivity disorder. Nat Genet. 2019;51(1):63-75.

9. Hammerschlag AR, Stringer S, de Leeuw CA, Sniekers S, Taskesen E, Watanabe K, et al. Genome-wide association analysis of insomnia complaints identifies risk genes and genetic overlap with psychiatric and metabolic traits. Nat Genet. 2017;49(11):1584-92.

10. Consortium SWGotPG. Biological insights from 108 schizophrenia-associated genetic loci. Nature. 2014;511(7510):421-7.

11. Consortium C-DGotPG. Identification of risk loci with shared effects on five major psychiatric disorders: a genome-wide analysis. Lancet. 2013;381(9875):1371-9.

12. Grove J, Ripke S, Als TD, Mattheisen M, Walters RK, Won H, et al. Identification of common genetic risk variants for autism spectrum disorder. Nat Genet. 2019;51(3):431-44.

13. Nagel M, Jansen PR, Stringer S, Watanabe K, de Leeuw CA, Bryois J, et al. Meta-analysis of genome-wide association studies for neuroticism in 449,484 individuals identifies novel genetic loci and pathways. Nat Genet. 2018;50(7):920-7.

14. Group PGCBDW. Large-scale genome-wide association analysis of bipolar disorder identifies a new susceptibility locus near ODZ4. Nat Genet. 2011;43(10):977-83.

15. Sniekers S, Stringer S, Watanabe K, Jansen PR, Coleman JRI, Krapohl E, et al. Genome-wide association meta-analysis of 78,308 individuals identifies new loci and genes influencing human intelligence. Nat Genet. 2017;49(7):1107-12.

16. Rietveld CA, Medland SE, Derringer J, Yang J, Esko T, Martin NW, et al. GWAS of 126,559 individuals identifies genetic variants associated with educational attainment. Science. 2013;340(6139):1467-71.

17. Okbay A, Baselmans BM, De Neve JE, Turley P, Nivard MG, Fontana MA, et al. Genetic variants associated with subjective well-being, depressive symptoms, and neuroticism identified through genome-wide analyses. Nat Genet. 2016;48(6):624-33.

18. Watanabe K, Taskesen E, van Bochoven A, Posthuma D. Functional mapping and annotation of genetic associations with FUMA. Nature Communications. 2017;8.

19. Võsa U, Claringbould A, Westra H-J, Bonder MJ, Deelen P, Zeng B, et al. Unraveling the polygenic architecture of complex traits using blood eQTL metaanalysis. bioRxiv. 2018:447367.

20. de Leeuw CA, Mooij JM, Heskes T, Posthuma D. MAGMA: generalized gene-set analysis of GWAS data. PLoS Comput Biol. 2015;11(4):e1004219.

21. Benjamini Y, Hochberg Y. Controlling the False Discovery Rate - a Practical and Powerful Approach to Multiple Testing. J Roy Stat Soc B Met. 1995;57(1):289-300.

**Supplementary Figure 1.** Q-Q plots. A, Analysis of PTSD cases; B, Analysis of PTSD symptom score.

**
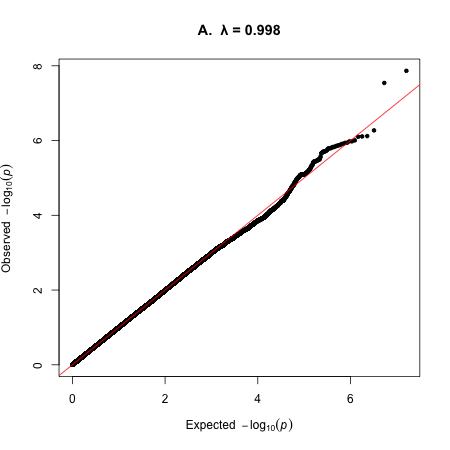

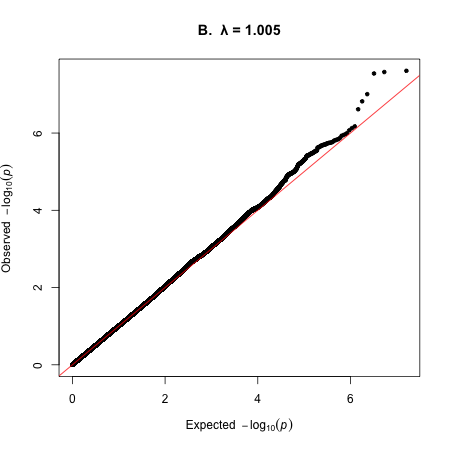
**

**Supplementary Figure 2.** Manhattan plot from the GWAS of PTSD cases in the CCSS cohort with additional adjustment for clinical characteristics and socioeconomic status. CCSS, Childhood Cancer Survivor Study; GWAS, genome-wide association study; PTSD, posttraumatic stress disorder. This plot displays 671 cases and 3,313 controls. Highlighted single-nucleotide polymorphisms were the lead ones and those within the same loci identified in Figure 1.

**
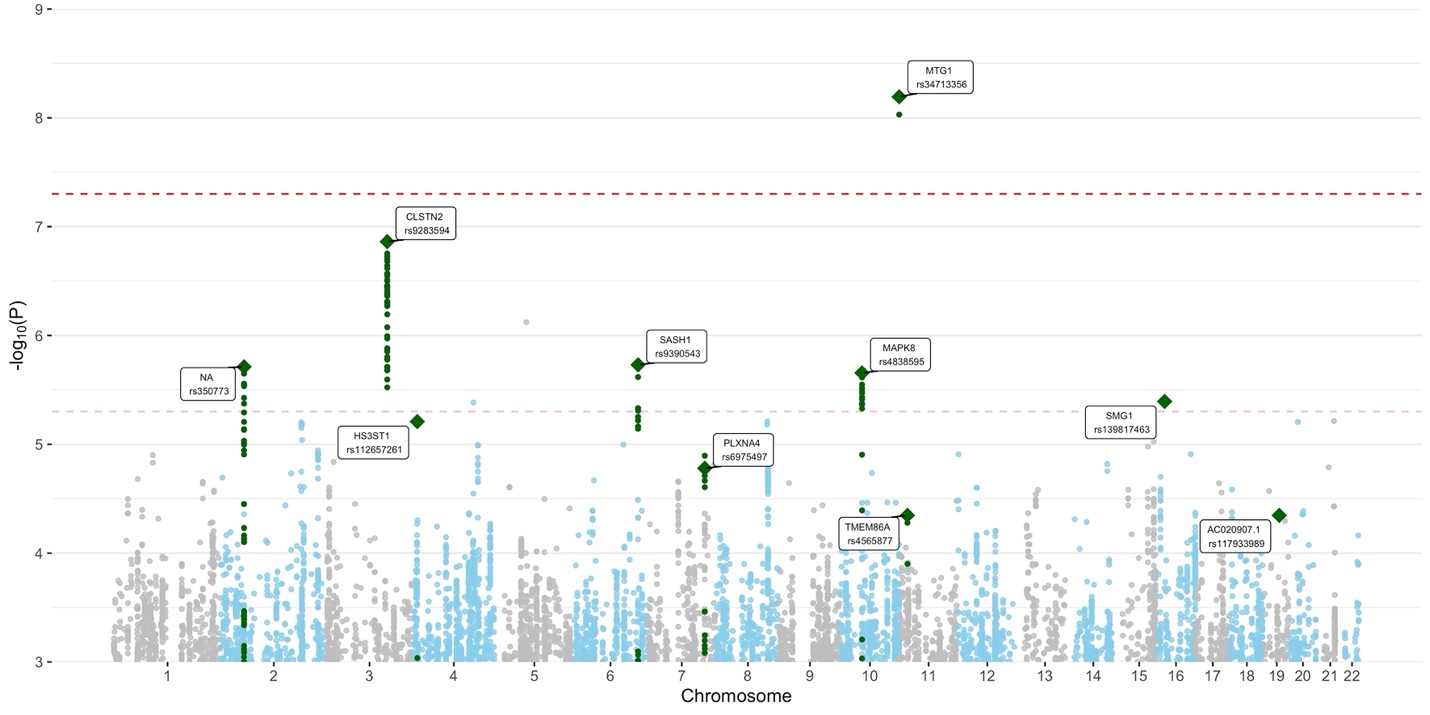
**

**Supplementary Figure 3.** Manhattan plot from the GWAS of PTSD symptom score (z-score), showing the top variants in 15 independent risk loci. GWAS, genome-wide association study; PTSD, posttraumatic stress disorder. This plot displays 3981 individuals. Single-nucleotide polymorphisms in green are in linkage disequilibrium (r^2^<0.1) with the index single-nucleotide polymorphisms (diamonds) and have a P value < 0.001. Index variants located with a distance less than 400 kilobase are considered as 1 locus. The model was adjusted for sex and top 10 principal components.


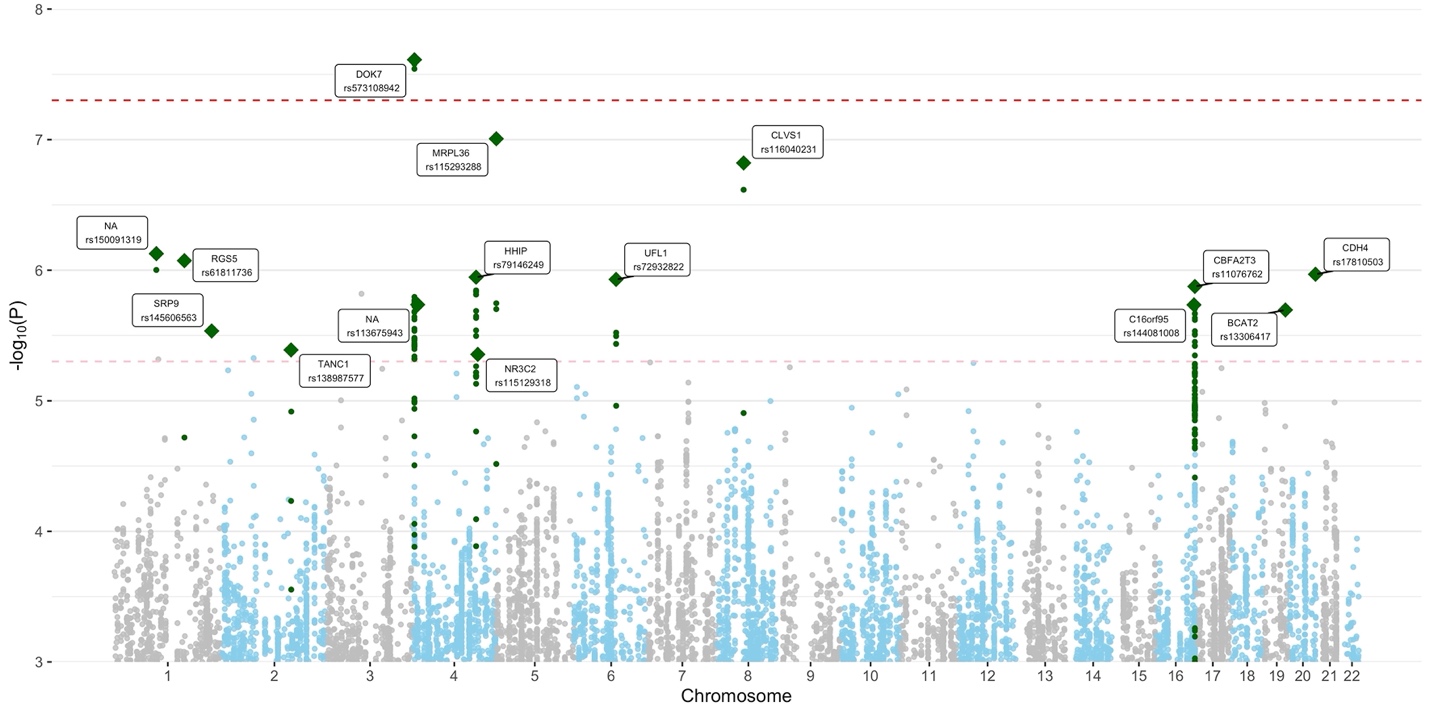


**Supplementary Figure 4.** Regional association of locus 10q26.3 anchored by the lead SNP rs34713356 with PTSD in cancer survivors (CCSS), PTSD in general population (PGC), and stress-related disorders (iPSYCH). CCSS, Childhood Cancer Survivor Study; iPSYCH, The Lundbeck Foundation Initiative for Integrative Psychiatric Research; PGC, Psychiatric Genomics Consortium; PTSD, posttraumatic stress disorder; SNP, single-nucleotide polymorphism.


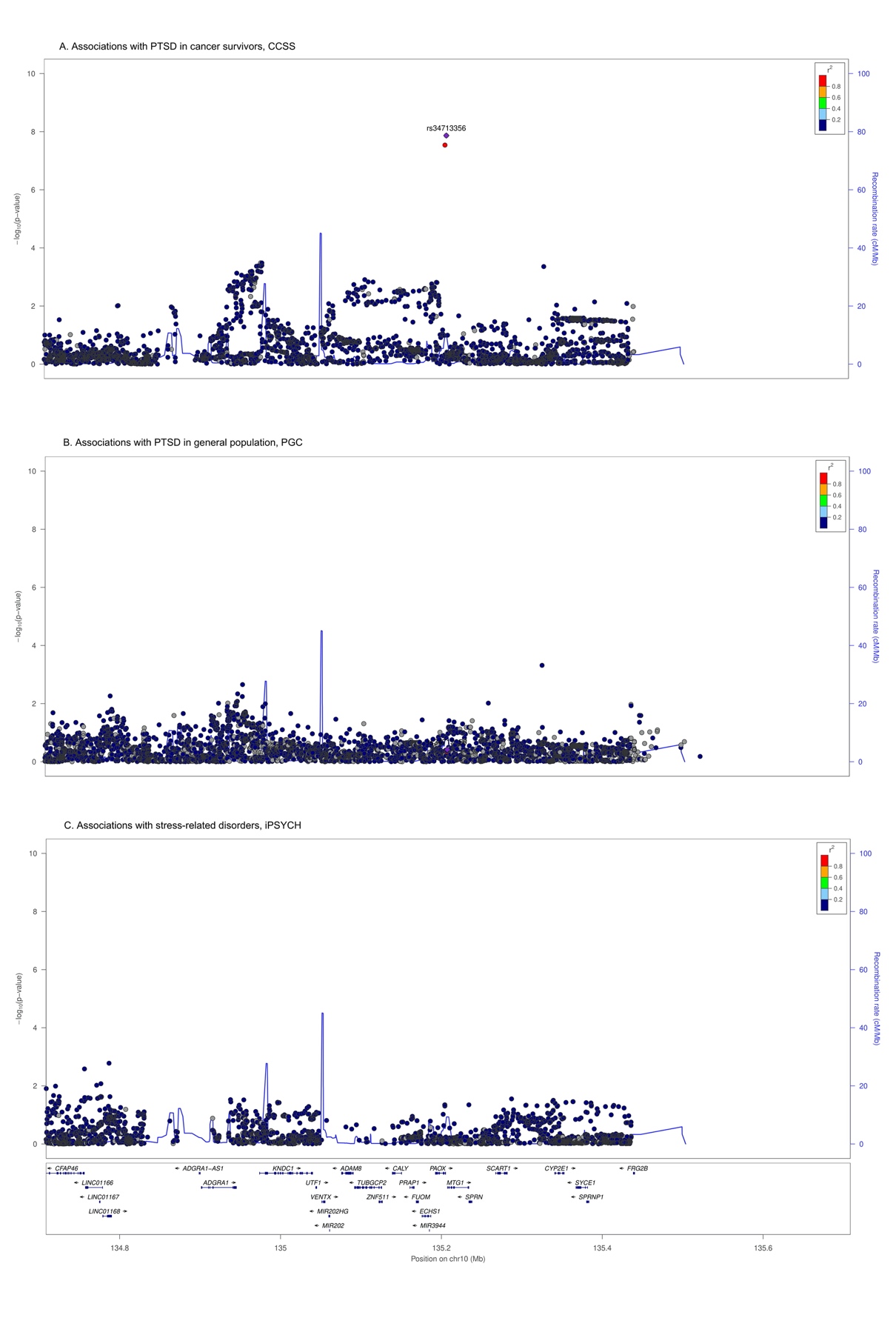


**Supplementary Figure 5.** Regional association of locus 6q24.3-q25.1 anchored by the lead SNP rs9390543 with PTSD in cancer survivors (CCSS), PTSD in general population (PGC), and stress-related disorders (iPSYCH). CCSS, Childhood Cancer Survivor Study; iPSYCH, The Lundbeck Foundation Initiative for Integrative Psychiatric Research; PGC, Psychiatric Genomics Consortium; PTSD, posttraumatic stress disorder; SNP, single-nucleotide polymorphism.


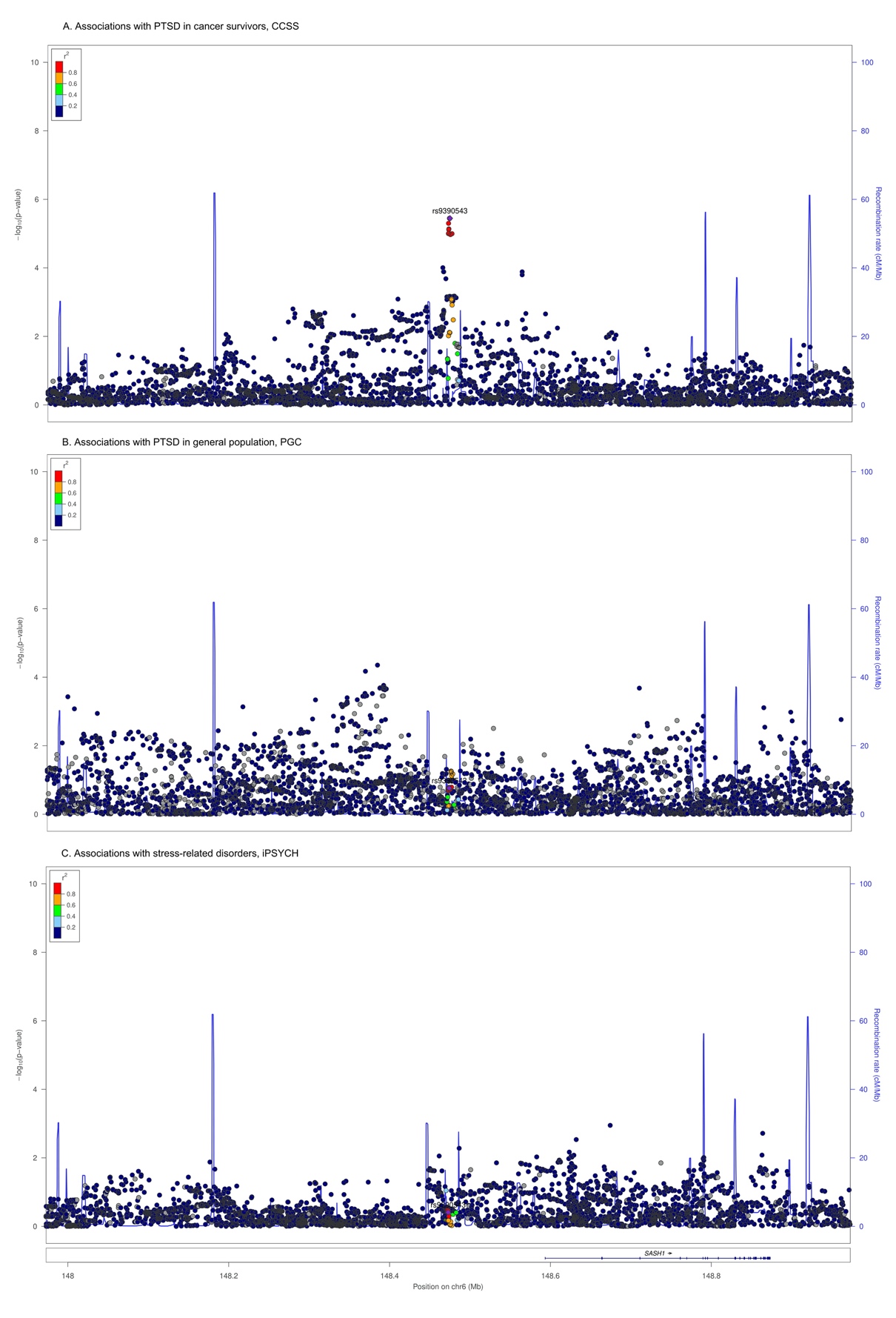


**Supplementary Table 1.** Risk of PTSD by genotype of the top variants among CCSS participants.

| rs34713356 | | | rs9390543 | | |
| --- | --- | --- | --- | --- | --- |
|  | Individuals, N | PTSD, N (%) |  | Individuals, N | PTSD, N (%) |
| GG | 2,957 | 443 (15.0) | AA | 1,181 | 242 (20.5) |
| GA | 868 | 191 (22.0) | GA | 1,971 | 320 (16.2) |
| AA | 83 | 24 (28.9) | GG | 818 | 107 (13.1) |

**Supplementary Table 2.** Lead SNPs in loci marginally associated with PTSD cases in the discovery cohort (CCSS) and their results in the replication cohort (SJLIFE) ^a^.

|  |  |  |  |  |  |  |  | Model 1 | | Model 2 | |
| --- | --- | --- | --- | --- | --- | --- | --- | --- | --- | --- | --- |
| Chr | Position | SNP | Gene | A1 | A2 | Sample | RAF | OR (95% CI) | P | OR (95% CI) | P |
| 3 | 139889148 | rs9283594 | *CLSTN2* | T | C | CCSS | 0.17 | 1.46 (1.26-1.69) | 7.60×10^-7^ | 1.51 (1.30-1.76) | 1.37×10^-7^ |
|  |  |  |  |  |  | SJLIFE | 0.20 | 1.04 (0.77-1.40) | 0.803 | 1.03 (0.75-1.41) | 0.847 |
|  |  |  |  |  |  | Meta-analysis | - | 1.36 (1.20-1.56)^*^ | 3.89×10^-6^ | 1.40 (1.22-1.61)^*^ | 1.45×10^-6^ |
| 16 | 19106269 | rs139817463 | *SMG1* | T | C | CCSS | 0.02 | 2.43 (1.70-3.48) | 1.15×10^-6^ | 2.41 (1.66-3.50) | 4.04×10^-6^ |
|  |  |  |  |  |  | SJLIFE | 0.02 | 1.08 (0.49-2.39) | 0.848 | 1.10 (0.48-2.56) | 0.818 |
|  |  |  |  |  |  | Meta-analysis | - | 2.12 (1.53-2.94) | 6.17×10^-6^ | 2.12 (1.50-2.98) | 1.66×10^-5^ |
| 2 | 52884802 | rs350773 | *-* | C | T | CCSS | 0.28 | 1.37 (1.21-1.56) | 1.37×10^-6^ | 1.38 (1.21-1.57) | 1.94×10^-6^ |
|  |  |  |  |  |  | SJLIFE | 0.26 | 1.04 (0.81-1.35) | 0.741 | 0.89 (0.67-1.18) | 0.408 |
|  |  |  |  |  |  | Meta-analysis | - | 1.30 (1.16-1.46) | 3.78×10^-6^ | 1.27 (1.13-1.44) | 7.32×10^-5^ |
| 7 | 132336527 | rs6975497 | *PLXNA4* | G | A | CCSS | 0.19 | 1.40 (1.22-1.61) | 2.94×10^-6^ | 1.38 (1.19-1.59) | 1.66×10^-5^ |
|  |  |  |  |  |  | SJLIFE | 0.20 | 1.02 (0.77-1.35) | 0.909 | 1.04 (0.76-1.41) | 0.816 |
|  |  |  |  |  |  | Meta-analysis | - | 1.32 (1.16-1.49)^*^ | 1.29×10^-5^ | 1.31 (1.15-1.49) | 6.41×10^-5^ |
| 4 | 11285914 | rs112657261 | *HS3ST1* | T | C | CCSS | 0.03 | 1.94 (1.47-2.56) | 3.26×10^-6^ | 1.95 (1.46-2.60) | 6.18×10^-6^ |
|  |  |  |  |  |  | SJLIFE | 0.03 | 1.03 (0.55-1.95) | 0.925 | 1.00 (0.50-2.00) | 1.000 |
|  |  |  |  |  |  | Meta-analysis | - | 1.75 (1.36-2.26) | 1.50×10^-5^ | 1.76 (1.35-2.30) | 3.04×10^-5^ |
| 19 | 35771995 | rs117933989 | *AC020907.1* | T | G | CCSS | 0.01 | 2.97 (1.88-4.71) | 3.46×10^-6^ | 2.71 (1.68-4.37) | 4.50×10^-5^ |
|  |  |  |  |  |  | SJLIFE | 0.01 | 1.71 (0.57-5.13) | 0.341 | 1.14 (0.33-3.99) | 0.838 |
|  |  |  |  |  |  | Meta-analysis | - | 2.74 (1.79-4.17) | 2.96×10^-6^ | 2.43 (1.55-3.80) | 1.03×10^-4^ |
| 11 | 18881631 | rs4565877 | *TMEM86A* |  |  | CCSS | 0.19 | 1.40 (1.21-1.61) | 3.66×10^-6^ | 1.35 (1.17-1.57) | 4.50×10^-5^ |
|  |  |  |  |  |  | SJLIFE | 0.18 | 0.89 (0.66-1.22) | 0.471 | 0.88 (0.63-1.23) | 0.441 |
|  |  |  |  |  |  | Meta-analysis | - | 1.29 (1.13-1.47)^*^ | 1.57×10^-4^ | 1.26 (1.11-1.44)^*^ | 5.97×10^-4^ |
| 10 | 49675247 | rs4838595 | *MAPK8* | T | C | CCSS | 0.12 | 1.48 (1.25-1.74) | 4.29×10^-6^ | 1.52 (1.28-1.80) | 2.21×10^-6^ |
|  |  |  |  |  |  | SJLIFE | 0.12 | 0.99 (0.69-1.43) | 0.965 | 0.97 (0.65-1.45) | 0.892 |
|  |  |  |  |  |  | Meta-analysis | - | 1.38 (1.18-1.61) | 3.97×10^-5^ | 1.41 (1.21-1.66)^*^ | 1.76×10^-5^ |

A1, Risk allele; A2, reference allele; CCSS, Childhood Cancer Survivor Study; Chr, chromosome; CI, confidence interval; OR, odds ratio; PTSD, posttraumatic stress disorder; RAF, risk allele frequency; SJLIFE, St. Jude Lifetime Study; SNP, single-nucleotide polymorphism.

^a^ Index variants are linkage disequilibrium independent (r^2^ <0.1) and are merged into 1 locus when located with a distance less than 400 kilobases. Genes were mapped in either positional (i.e., SNPs physically located inside a gene with up to 200 kilobase windows) or eQTL mapping (based on brain and blood samples from GTEx project as described previously).

^b^ Estimates were adjusted for sex and top 10 principal components.

^c^ Estimates were additional adjusted for age at cancer diagnosis, cancer type, surgery, chemotherapy, radiotherapy, educational level, employment status, personal income, and marital status.

^*^ P for heterogeneity <0.05.

**Supplementary Table 3.** Sensitivity analyses on rs34713356 and rs9390543 in the Childhood Cancer Survivor Study.

|  | rs34713356 | | rs9390543 | |
| --- | --- | --- | --- | --- |
| Associations with PTSD cases | OR (95% CI) | P | OR (95% CI) | P |
| With no depression/anxiety | 1.66 (1.35-2.04) | 1.70E-06 | 0.72 (0.61-0.84) | 4.15E-05 |
| With functional impairment or significant distress ^a^ | 1.41 (1.15-1.73) | 9.84E-04 | 0.74 (0.64-0.87) | 2.46E-04 |
|  |  |  |  |  |
| Associations with symptom score | β (95% CI) | P | β (95% CI) | P |
| Overall | 0.14 (0.07-0.20) | 2.19E-05 | -0.05 (-0.09-0.00) | 0.032 |
| Arousal | 0.11 (0.04-0.17) | 1.11E-03 | -0.04 (-0.08-0.01) | 0.087 |
| Avoidance | 0.14 (0.07-0.20) | 2.39E-05 | -0.04 (-0.08-0.01) | 0.097 |
| Re-experiencing | 0.10 (0.04-0.17) | 1.72E-03 | -0.06 (-0.10--0.02) | 7.83E-03 |

OR, odds ratio; PTSD, posttraumatic stress disorder; SES, socioeconomic status; SNP, single-nucleotide polymorphism.

^a^ An additional criteria was applied to assess the functional impairment and significant distress. Functional impairment was defined as a T-score <40 on the “role limitation due to emotional health” factor from the RAND Health Status Survey, Short Form-36 (SF-36). Significant distress was defined as a T-score >63 on the Global Status Index scale from the Brief Symptom Inventory-18 (BSI-18) or a T-score >63 on any 2 of 3 BSI-18 factors including Depression, Anxiety, and Somatization. In total, we identified 360 cases using this definition.

**Supplementary Table 4.** Lead SNPs in top loci associated with PTSD symptom score (z-score) ^a^.

|  |  |  |  |  |  |  | Discover: CCSS | | Replication: SJLIFE | | Meta-analysis | |
| --- | --- | --- | --- | --- | --- | --- | --- | --- | --- | --- | --- | --- |
| Chr | Position | SNP | Gene | A1 | A2 | RAF | β (95% CI) | P | β (95% CI) | P | β (95% CI) | P |
| 4 | 3648746 | rs573108942 | *DOK7* | T | C | 0.02 | 0.51 (0.33-0.69) | **2.45E-08** | -0.05 (-0.30-0.21) | 0.718 | 0.32 (0.18-0.47)^*^ | 1.65E-05 |
| 5 | 1756309 | rs115293288 | *MRPL36* | A | G | 0.02 | 0.48 (0.30-0.65) | 9.85E-08 | -0.03 (-0.33-0.27) | 0.852 | 0.35 (0.19-0.50)^*^ | 1.16E-05 |
| 8 | 62305844 | rs116040231 | *CLVS1* | A | G | 0.03 | 0.37 (0.23-0.51) | 1.51E-07 | 0.04 (-0.18-0.27) | 0.694 | 0.28 (0.16-0.40)^*^ | 4.23E-06 |
| 1 | 98756569 | rs150091319 |  | C | T | 0.02 | 0.41 (0.25-0.57) | 7.47E-07 | -0.01 (-0.26-0.24) | 0.943 | 0.29 (0.15-0.42)^*^ | 3.01E-05 |
| 1 | 163348893 | rs61811736 | *RGS5* | C | T | 0.01 | 0.47 (0.29-0.66) | 8.45E-07 | -0.25 (-0.57-0.07) | 0.133 | 0.30 (0.14-0.46)^*^ | 1.91E-04 |
| 20 | 60317327 | rs17810503 | *CDH4* | T | C | 0.03 | 0.30 (0.18-0.42) | 1.07E-06 | -0.04 (-0.23-0.15) | 0.673 | 0.20 (0.10-0.30)^*^ | 9.46E-05 |
| 4 | 145541236 | rs79146249 | *HHIP* | T | C | 0.02 | 0.41 (0.25-0.58) | 1.13E-06 | 0.10 (-0.15-0.35) | 0.439 | 0.32 (0.19-0.46)^*^ | 3.25E-06 |
| 6 | 96919173 | rs72932822 | *UFL1* | C | T | 0.06 | 0.24 (0.14-0.34) | 1.18E-06 | 0.16 (0.00-0.31) | **0.047** | 0.21 (0.13-0.30) | 4.95E-07 |
| 16 | 89162790 | rs11076762 | *CBFA2T3* | C | T | 0.26 | -0.12 (-0.17--0.07) | 1.33E-06 | 0.01 (-0.07-0.10) | 0.724 | -0.08 (-0.13--0.04)^*^ | 1.21E-04 |
| 4 | 11081758 | rs113675943 |  | T | C | 0.04 | 0.27 (0.16-0.39) | 1.84E-06 | 0.03 (-0.16-0.21) | 0.782 | 0.21 (0.11-0.30)^*^ | 1.81E-05 |
| 16 | 87123329 | rs144081008 | *C16orf95* | T | G | 0.02 | 0.44 (0.26-0.61) | 1.84E-06 | -0.07 (-0.36-0.23) | 0.654 | 0.30 (0.15-0.45)^*^ | 1.19E-04 |
| 19 | 49485075 | rs13306417 | *BCAT2* | T | C | 0.04 | -0.28 (-0.40--0.17) | 2.02E-06 | -0.01 (-0.24-0.22) | 0.924 | -0.22 (-0.33--0.12)^*^ | 4.20E-05 |
| 1 | 226154150 | rs145606563 | *SRP9* | G | A | 0.02 | 0.43 (0.25-0.60) | 2.92E-06 | 0.16 (-0.11-0.43) | 0.240 | 0.35 (0.20-0.49) | 5.65E-06 |
| 2 | 159645122 | rs138987577 | *TANC1* | C | G | 0.02 | 0.42 (0.24-0.60) | 4.08E-06 | -0.04 (-0.36-0.28) | 0.803 | 0.31 (0.15-0.46)^*^ | 1.15E-04 |
| 4 | 149428858 | rs115129318 | *NR3C2* | A | C | 0.01 | 0.52 (0.30-0.74) | 4.41E-06 | -0.12 (-0.81-0.56) | 0.744 | 0.46 (0.25-0.67)^*^ | 1.58E-05 |

A1, Risk allele; A2, reference allele; Chr, chromosome; CI, confidence interval; N sig., number of SNPs of P<0.05 in the locus; PTSD, posttraumatic stress disorder; RAF, risk allele frequency; SNP, single-nucleotide polymorphism.

^a^ Index variants are linkage disequilibrium independent (r^2^ < 0.1) and are merged into 1 locus when located with a distance less than 400 kilobases. Genes were mapped in either positional (i.e., SNPs physically located inside a gene with up to 200 kilobase windows) or eQTL mapping (based on brain and blood samples from GTEx project as described previously). Estimates were adjusted for sex and top 10 princip components.

^*^ P for heterogeneity <0.05.

**Supplementary Table 5.** Significant pathways identified in MAGMA gene-set analysis of PTSD cases.

| **Pathway** | **Ontology** | **N of genes** | **Beta** | **SE** | **P_empirical_** | **P_adjusted_** |
| --- | --- | --- | --- | --- | --- | --- |
| Arf guanyl nucleotide exchange factor activity | GO molecular function | 15 | 0.67 | 0.10 | 1.96E-11 | 2.98E-07 |
| Arf protein signal transduction | GO biological process | 15 | 0.58 | 0.10 | 2.50E-09 | 3.80E-05 |
| T helper 1 cell cytokine production | GO biological process | 3 | 1.20 | 0.22 | 1.97E-08 | 2.99E-04 |
| Regulation of programmed necrotic cell death | GO biological process | 9 | 0.49 | 0.09 | 6.16E-08 | 9.36E-04 |
| 4 hydroxyproline metabolic process | GO biological process | 5 | 0.82 | 0.16 | 2.00E-07 | 0.003 |
| Procollagen proline dioxygenase activity | GO molecular function | 3 | 0.88 | 0.17 | 2.34E-07 | 0.004 |
| Reactome interleukin 18 signaling | Curated gene sets | 3 | 0.96 | 0.20 | 4.68E-07 | 0.007 |
| Peptidyl proline hydroxylation | GO biological process | 6 | 0.72 | 0.15 | 4.78E-07 | 0.007 |
| Peptidyl proline dioxygenase activity | GO molecular function | 6 | 0.72 | 0.15 | 4.78E-07 | 0.007 |
| Response to interleukin 18 | GO biological process | 5 | 0.84 | 0.17 | 5.05E-07 | 0.008 |
| Peptidyl proline hydroxylation to 4 hydroxy l proline | GO biological process | 4 | 0.87 | 0.18 | 8.51E-07 | 0.013 |
| Peptidyl proline 4 dioxygenase activity | GO molecular function | 4 | 0.87 | 0.18 | 8.51E-07 | 0.013 |
| Pid arf6 pathway | Curated gene sets | 26 | 0.33 | 0.07 | 1.06E-06 | 0.016 |
| Programmed necrotic cell death | GO biological process | 22 | 0.35 | 0.07 | 1.20E-06 | 0.018 |
| Hydrolase activity acting on ether bonds | GO molecular function | 7 | 0.53 | 0.11 | 1.28E-06 | 0.019 |
| Interleukin 18 mediated signaling pathway | GO biological process | 4 | 0.87 | 0.19 | 2.16E-06 | 0.033 |

GO, Gene Ontology; MAGMA, Multi-marker Analysis of GenoMic Annotation; N of genes, number of significant genes identified in the pathway; P_empirical_, empirical P value; P_adjusted_, Bonferroni-corrected P value; PTSD, posttraumatic stress disorder; SE, standard error.
